# Patient Factors Predicting Weight Loss after Roux-en-Y Gastric Bypass

**DOI:** 10.1155/2017/3278751

**Published:** 2017-05-17

**Authors:** Linda Sillén, Ellen Andersson

**Affiliations:** Department of Surgery and Department of Clinical and Experimental Medicine, Linköping University, Norrköping, Sweden

## Abstract

**Objective:**

The purpose of this study was to identify preoperative factors predicting weight loss following Roux-en-Y gastric bypass (RYGB) surgery.

**Material and Methods:**

281 patients subjected to RYGB between January 2006 and June 2012 were included. Demographic, physical, and socioeconomic factors were assessed with regression analysis. Dependent variable was percent of excess weight loss (% EWL) at follow-up.

**Results:**

Follow-up data at one year was available in 96%, at two years in 88%, and at three years in 65% of the patients. Mean EWL was 72.5%. The success rate (defined as ≥60% EWL) at 1 year was 73% and at 2 years 74% and was 71% after 3 years. An earlier onset of obesity and high preoperative BMI were independently associated with unsuccessful weight loss at 1-year follow-up. At 2-year follow-up, an association between unsuccessful weight loss and psychiatric disorder, diabetes, hypertension, and preoperative BMI was seen. At 3-year follow-up no statistically significant associations were detected.

**Conclusions:**

RYGB provides successful weight loss for most patients. The results from this study indicate that an earlier age of onset of obesity, high preoperative BMI, psychiatric disorder, diabetes, and hypertension are associated with unsuccessful weight loss.

## 1. Introduction

Treatment of obesity is a global challenge and bariatric surgery is the most effective intervention to achieve and maintain substantial weight loss [1–3].

The most common bariatric procedure worldwide, the Roux-en-Y gastric bypass (RYGB), is associated with overall successful weight loss and positive results on comorbidity. It is important to stress that all patients do not lose weight successfully, despite precise surgical technique and regular follow-up [4, 5]. To be able to individually tailor treatment for the obese patient, it is of importance to learn more about the predictors related to outcome after bariatric surgery.

In previous studies, several factors have been associated with poor weight loss after bariatric surgery, such as male gender [5], older age [6, 7], being married [8, 9], greater initial weight and higher BMI [5–10], diabetes mellitus [5, 8, 10–12], psychiatric disorders [11, 13], reflux disease [14], and poor follow-up after surgery [15]. Lower educational level, unemployment, and lack of support are other risk factors that may predict poor outcome [9].

The purpose of this study was to identify preoperative patient related factors predicting unsuccessful weight loss following RYGB surgery.

## 2. Materials and Methods

The study was performed on all patients undergoing RYGB in one hospital in southern Sweden (Vrinnevi Hospital, Norrköping) between January 2006 and June 2012. Data was collected through a patient questionnaire completed by all patients referred to the department of surgery for RYGB and through patients' charts. Follow-up data were collected from the Scandinavian Obesity Surgery Registry (SOReg) and patients' charts. The information collected in the patient questionnaire was confirmed at a preoperative multidisciplinary team evaluation, including a complete medical history, physical examination, biometric measurements, laboratory workup, and psychological and nutritional evaluations and going through the patients' charts. The multidisciplinary team consisted of a bariatric surgeon, a dietitian, a physical therapist, a counsellor, and a nurse. Prerequisites to be considered suitable for a RYGB procedure included a high degree of patient motivation and capacity to understand the postoperative, lifelong regimens. Ongoing eating disorders and severe, untreated psychiatric disorders were considered exclusion criteria. All patients were put on a preoperative low caloric diet, with a mandatory preoperative loss of at least 5% of the initial weight. Open operations were the standard procedure during the early years, while laparoscopic operations have been predominating in the last few years. After surgery patients had appointments with members of the same medical team after six weeks and one year and then at primary health care centers annually.

Written consent from the patients was obtained when filling out the questionnaire.

Those responsible for the SOReg registry and the Clinical Director were contacted for permission to use the clinic's registry data. All study procedures were approved by the Regional Ethics Review Board in Linköping, Sweden, Dnr 2013/30-31.

### 2.1. Study Variables

Information collected in the questionnaire and from follow-up included demographic, socioeconomic, and clinical variables including age, gender, smoking (current/never/previous smoker) and drinking habits (number of weekly occasions when alcohol was consumed), highest level of education (elementary school/high school/university degree), marital status (married/single), employment (yes/no), preoperative weight and BMI, and onset of obesity (childhood/adolescence/adult). The prevalence of obesity-associated comorbidities (hypertension, type 2 diabetes, and hyperlipidemia) and a previous history of psychiatric disorders were based on the patients' self-reported medical history and confirmed by going through patients' charts. The comorbidities were reported as either present or absent.

Most of the questions were categorical questions with dichotomous response.

To evaluate outcome, percentage of excess weight loss (% EWL) was used. An EWL ≤ 60% was chosen as the cutoff to characterize unsuccessful weight loss.

### 2.2. Statistical Analysis

Frequencies and descriptive statistics were calculated for all variables, using mean, standard deviation (SD) for continuous variables and frequencies and percentage for categorical variables. For comparison* t*-test was used for continuous variables and chi-square (*χ*^2^) for categorical variables.

General linear regression was used to assess independent associations between baseline variables and EWL at follow-up. Binary logistic regression was used in multivariate analysis to identify independent preoperative variables associated with unsuccessful weight loss, defined as EWL ≤ 60%. Statistical significance was set to *P* < 0.05. All statistical analyses were performed using SPSS (IBM® SPSS® Statistics 22).

## 3. Results

During the study period 338 patients had a RYGB procedure. Exclusion criteria were patients lost to one-year follow-up and patients scheduled for a revisional RYGB procedure, as the indication for surgery may be different from a primary RYGB operation. Based on these criteria the final study population consisted of 281 patients. Follow-up data at one year was available in 96% of cases and in 88% and 65% at two and three years, respectively.

Demographic data and preoperative comorbidities for all patients who completed one-year follow-up are presented in Table 1.

The mortality rate was 0.5% (2 patients). One of the deceased patients died by heart failure after a reoperation at the fourth postoperative day, because of a massive bleeding from the gastroenteroanastomosis. The second patient committed suicide 22 months after surgery.

An open RYGB surgical approach was used in 160 patients (56,9%), while the rest had a laparoscopic procedure. No statistically significant difference in weight loss after surgery was detected between the laparoscopic and the open surgery groups.

A mean EWL% of over 70% was detected during follow-up until three years postoperatively. Outcome, concerning weight loss, is presented in Table 2.

The rate of diabetes remission after surgery was 56% at one-year follow-up, 63% after two years, and 66% after three years. The corresponding figures for hypertension were 39%, 41%, and 57% and for hyperlipidemia 54%, 34%, and 74%. Remission was defined as patients no longer requiring any antidiabetic, antihypertensive, or lipid-lowering agents.

**Figure 1:**
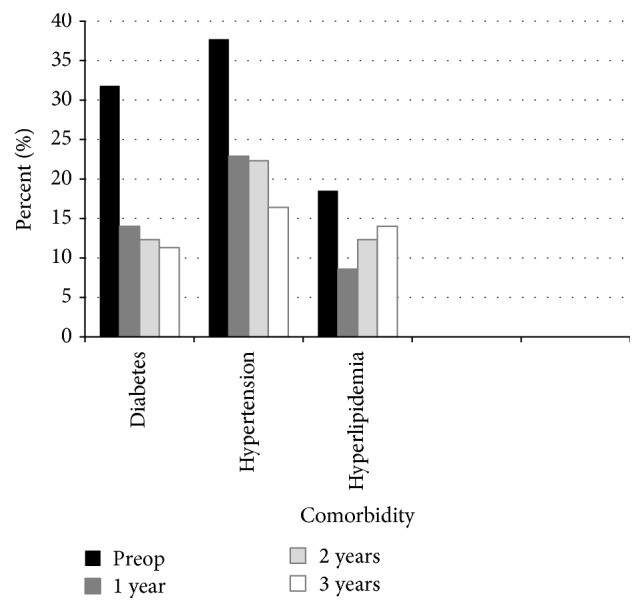
Demographic and postoperative clinical data of comorbidity.

Outcome, concerning comorbidities, is presented in Figure 1.

Binary logistic regression was used to identify independent preoperative factors associated with unsuccessful weight loss (EWL < 60%). An earlier age of onset of obesity and a higher preoperative BMI were independently associated with unsuccessful weight loss at one-year follow-up (Table 3).

Two-year follow-up data showed an association between psychiatric disorder, preoperative diabetes, preoperative hypertension, high preoperative BMI, and unsuccessful weight loss (Table 4).

At three-year follow-up no statistically significant associations were detected by the analysis.

## 4. Discussion

Compared to previous studies of weight loss outcome after RYGB, the majority of patients in our study achieved a successful weight loss, with a mean EWL of 73,6% (29–246%), and a total mean weight loss of 44 kg (14–144 kg) three years postoperatively. Defining successful weight loss as EWL ≥ 60% at one year after surgery, our group of patients had a 72% success rate, leaving 28% of patients with an unsuccessful result from a weight loss perspective. A previous study including both RYGB and sleeve gastrectomy patients with the same cutoff 60% EWL showed similar results, with a success rate of 74% [7].

In our study, preoperative factors associated with suboptimal weight loss at one year after RYGB included onset of obesity in childhood, greater preoperative weight, and a high initial BMI. These results are supported by previous studies showing that a greater preoperative weight and BMI are associated with poorer weight loss after RYGB [5, 6, 8, 10]. The underlying etiology may be a lower level of activity in heavier patients, but may also reflect genetic differences, and the fact that heavier patients, despite a large total weight loss, still may have a lower EWL compared to lighter patients [5, 6].

When it comes to onset of obesity, gender, marital status, and employment, the results are harder to interpret, as results from previous studies diverge. In our study patients with adulthood onset of obesity were more likely to achieve successful weight loss, compared to patients with obesity since childhood. This may be explained by an early onset of the metabolic syndrome and lifelong lack of habits of healthy eating and exercise. Childhood obesity is known to be associated with lifelong reduced physical activity and a risk of decreased self-esteem, depression, and social isolation, which may further augment the risk of failure to lose weight after surgery [16]. However, the opposite results have been showed in another study [17].

Gender, marital status, and employment status did not seem to affect weight loss in the present study. In accordance with most studies, the vast majority, 198/281 patients, were female. In other studies results are varying, with no gender differences and better results in single patients in one study [8], while others have reported greater EWL in women and in married patients [5, 17].

In this study, preoperative comorbidities and psychiatric disorder have a statistically significant effect on weight loss at two-year follow-up. Prevalence of self-reported psychiatric disorders was rather low, 21%, compared to earlier studies in RYGP patients with a rate of 55–60% [18, 19]. In several previous studies, psychiatric conditions have been associated with a poorer adherence to both pre- and postoperative medical instructions and insufficient postoperative weight loss [11, 13]. This is despite the fact that these patients are a selected group of patients, as patients with severe inability to adhere to preoperative programs or to show documented weight reduction may not to be considered candidates for surgery.

In accordance with prior studies, there is a large effect of RYGB on comorbidity. The diabetes remission rate three years after surgery was 65%, consistent with results from prior studies [20, 21]. Remission of hypertension, 56%, is higher than another study with a 38% remission rate [21] and comparable to the Utah Obesity Study of RYGB with 53% [20]. Hyperlipidemia was in remission in 74%, which is higher than other studies with rates of 53% and 59% [20, 21].

Several studies have shown that diabetes is associated with a lower EWL [5, 6, 11, 12]. Our results showed an association between lower EWL and preoperative diabetes and hypertension two years after surgery. After one year, however, no such association was seen. One reason may be that patients tend to fall back into preoperative eating patterns and habits of exercise two years after surgery, while still more adherent to lifestyle changes one year postoperatively.

Mean EWL in this study was high, 73%. It is higher than several other studies which have shown results of about 60% [3, 5, 6]. An explanation may be a successful selection of patients, thorough perioperative support and follow-up by a multidisciplinary team [22]. Adherence to nutritional guidelines postoperatively and the practice of physical activities are fundamental factors for sufficient weight loss maintenance over the years [11], and thorough follow-up may help the patients to adhere to these recommendations.

The present study has strengths and limitations. The relatively high number of patients enrolled in this study is a strength, as well as a prolonged follow-up period of three years.

There are, however, several limitations to this study. The study population comes from a single center, which may make the results less applicable in a wider perspective. Further, follow-up data at two and three years are not complete. The follow-up rate of 96% at one year is good, whereas 88% and 65% at two and three years postoperatively are more discouraging. A large Swedish study has reported weight changes over a 10-year period, with maximal weight loss after one year and some weight regain after two years [23]. Our results indicate a sustainable loss of weight during the three-year follow-up. However, one may argue that the patients with bad compliance to follow-up also may have worse results concerning weight loss. This may be true, but when comparing weight loss after one year, the patients not attending the two-year follow-up did not have an inferior weight loss compared to the patients attending the two-year follow-up.

In this study successful weight loss is defined as ≥60% EWL, which is a tougher cutoff than many other studies. The use of various outcome measures has often made comparison of trials difficult. Definition of successful weight loss varies among previous studies, with some using 40% EWL as a cutoff, [5, 12], whereas others use 50% EWL [6] and 60% EWL [7]. Perhaps a more important index of success is resolution of obesity related comorbidities and improvement in quality of life [8, 24]. Nevertheless, weight loss is the most easily measureable and frequently used parameter and a parameter that many patients consider to be very important. Considering patients' expectations on weight loss, studies indicate that patients are reported to expect a mean EWL of as much as 80–106% [25, 26].

## 5. Conclusion

In conclusion, our study shows that a high preoperative weight, high preoperative BMI, childhood obesity, psychiatric disorder, preoperative diabetes, and preoperative hypertension are independently associated with a risk of suboptimal weight loss after RYGB. These findings emphasize the need of being extraobservant on support of patients with these risk factors.

Patients suffering from obesity are a heterogeneous group of individuals, where much more knowledge is needed in the struggle to be able to tailor the optimal treatment for each person. Our results may add a small piece to this multidimensional puzzle.

## Figures and Tables

**Table 1:** Data expressed as *n* (%) for nominal variables or mean (range) for continuous variables.

Variable	Entire group (*n* = 281)
Gender	
Women	198 (70,4%)
Age (years)	40,8 (16–67)
Marital status	
Married	179 (64,2%)
Employed	144 (51,8%)
Retired	14 (5%)
Sick listed	63 (22,5%)
Student	23 (8,2%)
Unemployed	30 (10,7%)
Comorbidities	
Diabetes	93 (32%)
Hypertension	110 (37,7%)
Hyperlipidemia	54 (18,5%)
Gallstone	40 (14,4%)
Psychiatric disorder	61 (21,7%)
Start of obesity	
Childhood	99 (36,5%)
Adolescence	80 (29,5%)
Adulthood	92 (33,9%)
Preoperative weight (kg)	135,7 (85–254)
Preoperative BMI (kg/m^2^)	45,5 (31,9–80,7)

**Table 2:** Weight loss outcomes. Data are expressed as mean (range).

	Preop	1 year postop *n* = 281	2 years postop *n* = 247	3 years postop *n* = 182
Weight (kg)	135 (85–250)	92,0 (49–187)	90,1 (48–168)	91,7 (57–154)
BMI (kg/m^2^)	46,5 (31,9–80)	31,7 (18–60)	31,0 (21–54)	31,1 (21–50)
Weight loss (kg)		43,4 (11–111)	44,6 (12–128)	44,3 (14–144)
EWL%		72,6 (13–207)	73,9 (30–135)	73,6 (29–246)

**Table 3:** Logistic regression analysis correlating preoperative clinical factors and successful versus unsuccessful weight loss in patients following RYGB at 1-year follow-up. Data expressed as *n* (%) for nominal variables or mean for continuous variables. Successful EWL was defined as ≥60%; unsuccessful EWL ≤ 59%. ^b^Comparison of successful versus unsuccessful EWL. OR: odds ratio.

	Successful EWL (*n* = 203)	Unsuccessful EWL (*n* = 78)	*P* value^b^	OR
Men	57 (28%)	29 (37%)		
Women	146 (72%)	49 (63%)	0.112	1.6
Age (years)	40,9	41,03	0.228	1.3
Marital status				
Married	132 (66%)	47 (60%)		
Single	69 (34%)	31 (40%)	0.109	1.5
Employment	110 (55%)	34 (44%)	0.119	0.6
Diabetes	63 (31%)	27 (35%)	0.545	1.2
Hypertension	70 (35%)	33 (42%)	0.304	1.3
Hyperlipidemia	38 (19%)	11 (14%)	0.161	0.5
Psychiatric disorder	38 (19%)	21 (27%)	0.404	1.3
Gallstone	34 (17%)	6 (8%)	0.059	0.4
Onset of obesity				
Childhood	63 (32%)	36 (48%)	0.005	1.6
Adolescence	57 (29%)	23 (30%)		
Adulthood	75 (39%)	17 (22%)		
Diabetes, postop 1 year	25 (12%)	16 (20%)	0.331	1.4
Hypertension, postop 1 year	43 (21%)	24 (31%)	0.147	1.6
Hyperlipidemia, postop 1 year	18 (9%)	7 (9%)	0.417	0.6
Preoperative weight (kg)	130,9	147,6	<0.001	0.9
Preoperative BMI (kg/m^2^)	45,0	50,6	<0.001	0.8

**Table 4:** Logistic regression analysis correlating preoperative clinical factors and successful versus unsuccessful weight loss in patients following RYGB at 2-year follow-up. Only significant data are presented. Data expressed as *n* (%) for nominal variables or mean for continuous variables. Successful EWL was defined as ≥60%; suboptimal EWL ≤ 59%. ^b^Comparison of successful versus unsuccessful EWL. OR: odds ratio.

	Successful EWL (*n* = 182)	Unsuccessful EWL (*n* = 65)	*P* value^b^	OR
Psychiatric disorder	20 (31%)	38 (17%)	0.004	0.3
Diabetes, postop 2 years	19 (11%)	16 (25%)	0.027	0.2
Hypertension, postop 2 years	39 (22%)	23 (39%)	0.026	0.3
Preoperative BMI (kg/m^2^)	45,3	50,2	0.015	0.8
